# Unravelling the Bcl-2 Apoptosis Code with a Simple Model System

**DOI:** 10.1371/journal.pbio.0060154

**Published:** 2008-06-17

**Authors:** Gorka Basañez, J. Marie Hardwick

## Abstract

Using defined lipids and purified proteins, Billen et al. offer a new model to reconcile the two currently opposing models for how Bcl-2 family member interactions regulate cell death.

Every day, a healthy human loses billions of cells by design [1]. Cell death processes, such as apoptosis and other tightly regulated mechanisms that kill old, damaged, or unwanted cells, are critically important for normal embryonic development and for the maintenance of physiological functions in multicellular organisms (and likely in unicellular species as well) [1,2]. Insufficient or excessive cell death underlies most human pathologies, including cancer, autoimmunity, infectious diseases, and degenerative disorders. Thus, it is not surprising that efforts at identifying the factors responsible for cell death and elucidating their detailed mechanisms of action have received considerable research attention.

Much of this attention has focused on the Bcl-2 protein family, which has been recognized as the principal regulatory component of the intracellular apoptotic machinery for over two decades [3]. Bcl-2 family proteins can either inhibit or promote apoptosis, largely depending on the number of Bcl-2 homology (BH) domains they share. For example, anti-apoptotic family members, such as Bcl-2 and Bcl-XL, have four BH domains, while pro-apoptotic members such as Bax and Bak contain three, and a third subset, the BH3-only proteins, contains just one. Although there is growing interest in the newly identified roles that both pro- and anti-death Bcl-2 family proteins play in healthy cells—for example, in membrane fission and fusion mechanisms, mitochondrial autophagy, neuronal activity, and cellular energetics [3,4]—most current research is focused on the functions of this family during cell death. Though not universally accepted, it is widely thought that Bcl-2 family proteins regulate commitment to apoptosis primarily through their capacity to control the permeability of the outer mitochondrial membrane (OMM): permeabilization triggers the release of multiple apoptogenic factors into the cytosol and/or leads to mitochondrial dysfunction [5]. Various Bcl-2 family members affect this key event of the apoptotic cascade in different ways, determining their pro- or anti-apoptotic status. The Bcl-2-type proteins inhibit OMM permeabilization, thereby preserving cell viability. In contrast, Bax-type proteins and the diverse group of BH3-only proteins facilitate OMM permeabilization and thus promote cell death. Yet despite intense effort, the question of exactly how different types of Bcl-2 family proteins fulfil these tasks remains actively debated [3,5,6].

## The Bcl-2 Network

Part of the difficulty in resolving the functions of Bcl-2 family proteins is the inherent complexity of the cellular apoptotic network. An additional hurdle is that Bcl-2 protein action likely occurs at membranes, and membrane proteins can be made to reveal their secrets only with difficulty, as lipid-based systems are more challenging to study. Fortunately, basic aspects of the regulation of OMM permeability by Bcl-2 family proteins appear amenable to reconstitution in vitro, using simplified systems made up of purified proteins and lipids. As a prominent example, early studies from several laboratories using recombinant proteins and pure lipid membranes revealed that pro-apoptotic (activated) Bax has an intrinsic pore-forming activity [7–9]. These and other results obtained from reconstitution approaches were critical to establishing the currently popular view that the primary pro-death function of Bax-type proteins is direct permeabilization of the OMM. However, understanding exactly how Bcl-2-type proteins and BH3-only proteins regulate Bax permeabilizing function has proven more difficult.

Bax is often found as a monomeric protein in the cytosol of healthy cells. In cells committed to death, Bax appears to change conformation, translocate to the OMM, and assemble into intramembrane complexes [3]. BH3-only proteins funnel diverse apoptotic signals so as to trigger Bax conformational change and Bax-driven OMM permeabilization, but many details of this molecular pathway remain unclear. On the other hand, although essentially all Bcl-2 family proteins have been reported to bind a wide range of diverse protein partners, it has long been recognized that the ability of different Bcl-2 family members to engage in selective protein–protein interactions with other family members is integral to their anti- and pro-death functions [1]. Indeed, structural studies sustain the notion that an elongated hydrophobic groove on pro-survival proteins can act as a “receptor” binding-pocket for the amphipathic (that is, hydrophobic at one side and hydrophilic at the other) a-helical BH3 “ligand” of pro-apoptotic partners [10].

Largely based on these notions, two major competing models have been proposed (Figure 1). On the one hand, the “sensor-only BH3” or “displacement” model (also known as the “indirect” model for BH3-only proteins, and the “direct” model for Bcl-2 types) postulates that Bcl-2-type proteins directly bind to and neutralize Bax-type proteins. In turn, Bax-type proteins auto-activate when their anti-apoptotic Bcl-2 partner is displaced upon binding to a BH3-only protein [11]. One complication with this model is that mutants of Bcl-2-type proteins that fail to bind Bax and Bak can be fully protective against cell death. The alternative “split BH3” model (“direct” model for BH3-only, “indirect” model for Bcl-2 types) posits that Bax-type proteins are activated via interactions with a selective subset of activator-type BH3-only members, such as cleaved/truncated Bid (tBid), and that Bcl-2-type proteins act by sequestering these activator-type BH3-only proteins, rather than Bax-type proteins [12,13]. As in the sensor-only model, the sensor-type BH3-only proteins such as Bad can still inactivate Bcl-2-type proteins. A major complication with this model is that direct interactions between activator-type BH3-only proteins and Bax-type proteins are difficult to detect. There are other permutations of these two models, such as the “hierarchical model,” in which unrelated cell factors engage Bcl-2 family proteins to act at intermediate steps, alleviating the need for direct binding of BH3-only and Bax-type proteins [3,5,6]. One potential downside of all of these models is that many of the protein–protein interaction specificities were determined from solution-based studies in the absence of (or following extraction from) membranes, or with protein fragments instead of full-length proteins. Presumably the behavior of soluble Bcl-2 proteins and derived peptides will not fully reveal their mechanisms of action at the mitochondrial (or other) membrane locus.

**Figure 1:**
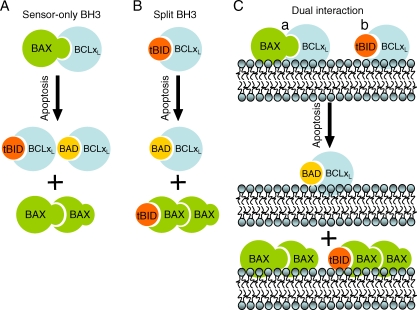
Schematic Models to Explain the Functional Relationships between Bcl-2 Family Proteins Depicted are the anti-death Bcl-2-type protein Bcl-XL, the pro-death protein Bax, and the BH3-only proteins Bid (N-terminally truncated Bid or tBid, an activator- or sensor-type), and Bad (sensor-type BH3-only protein). (A) In the sensor-only model, all BH3-only proteins serve to inactivate Bcl-2-like cell death inhibitors, which when released allow activation and oligomerization of Bax. (B) In the split BH3 model, some BH3-only proteins act upstream of Bcl-2-types, while others act downstream. The BH3-only protein tBid is sequestered by Bcl-XL, and when tBid is displaced by sensor-type BH3-only protein Bad, tBid is freed to activate Bax, which in turn oligomerizes in the membrane to facilitate release of pro-apoptotic proteins. (C) In the dual interaction model put forth by Billen and colleagues, Bcl-XL can interact with either Bax or tBid in the same sample.

## Dual Interaction Model

In this issue of *PLoS Biology*, Billen et al. elegantly address this problem using a powerful combination of approaches applied to in vitro reconstitution systems appropriately consisting of full-length purified proteins and relevant membrane targets (isolated mitochondria and pure lipid OMM-like vesicles, or liposomes) [14]. One important contribution of the study is the demonstration that Bcl-XL engages in stable interactions with both Bax and tBid, rather than with only one of the two pro-apoptotic molecules, as had been proposed in previous models [11–13]. Moreover, substantial correlation between mitochondria- and liposome-based systems was found, whereby Bcl-XL forms two different stable heterodimers, one with Bax, the other with tBid, and both of these interactions contribute to the neutralization of the Bax permeabilizing function. An exciting avenue for further research will be the development of assays in reconstituted systems that allow detection and monitoring of dynamic (rather than static) interactions between specific anti- and pro-apoptotic Bcl-2 family members plus additional cellular proteins in membranes.

Billen et al. [14] also provide important mechanistic insight by delineating three distinct actions of Bcl-XL that inhibit activation of Bax permeabilizing function. Toward this end, the investigators focused on studies using OMM-like liposomes. First, they show that Bcl-XL abrogates the transient association of Bax with the liposome surface that is necessary for shifting soluble Bax molecules into a conformation that is susceptible to productive interactions with tBid [15]. In addition, Bcl-XL directly competes with soluble Bax for binding to tBid, thereby preventing the tBid–Bax interactions that are required for effective recruitment of Bax to the liposome bilayer and for subsequent events leading to intramembranous Bax oligomerization. Finally, Bcl-XL also neutralizes homotypic Bax interactions that contribute to activation of the Bax permeabilizing function. Oligomerization perhaps involves the BH3 motif of one Bax molecule binding in the BH3 binding pocket on the opposite face of the next Bax molecule, as recently proposed for Bak [16]. Thus, in contrast to the previous linear models, this report provides the strongest evidence to date that Bcl-XL uses multiple mechanisms to neutralize functional Bax activation.

Another intriguing outcome of this study is the observation that interaction of tBid and/or activated Bax with Bcl-XL triggers translocation of Bcl-XL to the membrane. Structural studies with the Bcl-XL homologue Bcl-w [10] revealed that, like Bax, a C-terminal hydrophobic helix folds intramolecularly, occupying the same hydrophobic groove previously assumed to act only as the BH3-binding pocket. Once freed from the rest of the protein, the C-terminal hydrophobic helix functions as a transmembrane domain through which Bcl-2 family proteins can be anchored to the OMM. Therefore, one explanation is that engagement of the BH3 domain of tBid and/or activated Bax into the Bcl-XL hydrophobic groove displaces the C-terminal hydrophobic helix of Bcl-XL, allowing anchoring of the protein to the lipid bilayer.

## Future Directions

Notwithstanding these important contributions, the model presented in this study also raises fascinating new questions. Chief among these: what is the structure adopted by Bcl-XL in the membrane, and how does this structure explain the inhibition of Bax permeabilizing function? One possibility is that membrane-associated Bcl-XL adopts an overall structure similar to that found in solution, except with its C-terminal helix inserted into the lipid bilayer in a transmembrane orientation (Figure 2A and 2B). This would allow interactions of the Bcl-XL groove with BH3 domains from pro-apoptotic partners sequestering Bax and/or tBid at the membrane locus, either of which could result in neutralization of Bax-mediated membrane permeabilization.

**Figure 2:**
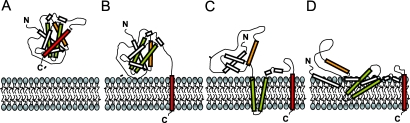
Putative Conformations of Bcl-XL with a-Helices 2 (BH3 Domain), 5–6 (Central Hairpin), and 9 (C-Terminal Hydrophobic Tail) Depicted in Orange, Green, and Red, Respectively (A) Structure of Bcl-XL in solution. (B) Membrane-integrated conformation of Bcl-XL in which the C-terminal tail is inserted into the membrane in a transmembrane orientation, while the remainder of the protein maintains the solution structure. In this conformation, the BH3-binding pocket of Bcl-XL is expected to be available to engage in inhibitory groove–BH3 interactions with pro-death Bcl-2 family proteins, such as tBid and Bax, as is seen in solution-based binding studies. (C) Membrane-integrated conformation of Bcl-XL in which the C-terminal tail and the central helical hairpin are inserted into the membrane. In this conformation, the BH3-binding pocket of soluble Bcl-XL is expected to be substantially modified or destroyed. However, Bcl-XL may still engage in neutralizing interactions with Bax, as previously proposed for Bcl-2 [20]. Alternatively, this conformation may correspond to functionally silent Bcl-XL, pro-apoptotic Bcl-XL, or a yet unknown function of Bcl-XL. (D) Membrane-integrated conformation of Bcl-XL, in which many different helical regions of the protein are inserted in the membrane, but with different orientations and at different penetration depths. In this conformation, the BH3-binding pocket of soluble Bcl-XL no longer exists. Nevertheless, it is conceivable that Bcl-XL inhibits pro-apoptotic proteins through interaction surfaces other than the groove and/or via interactions with membrane lipids. Alternatively, this conformation may correspond to functionally silent, pro-apoptotic, or a yet unknown function of Bcl-XL. We postulate that the topology adopted by Bcl-2-type proteins in the membrane can be modulated by changes in membrane lipid composition.

Alternative possibilities exist. Indeed, biochemical labeling of Bcl-2 and Bax in cells previously led to the conclusion that both proteins insert a central helical hairpin into the lipid bilayer of the OMM [17,18]. This conclusion is consistent with structural similarities found between Bcl-2- and Bax-type proteins and bacterial pore-forming toxins such as colicins and diphtheria toxin [10,19]. Moreover, this conformation was linked to the anti-apoptotic function of Bcl-2 due to neutralizing hairpin–hairpin interactions with Bax [20]. In line with this observation, biophysical studies with model membranes suggest that not only Bcl-2 but also Bcl-XL can insert central helical hairpins into the hydrophobic matrix of the lipid bilayer [21,22]. However, much remains to be learned concerning the orientation and depth of penetration within the lipid bilayer of helical hairpin segments of different Bcl-2 family members (Figure 2C and 2D). Regardless, it is important to point out that membrane insertion of the central helical hairpin almost certainly eliminates the BH3-binding pocket present in soluble structures of Bcl-2-type proteins. Additional controversies arise from studies relating membrane conformational changes of Bcl-2-type proteins with acquisition of a pro-apoptotic phenotype due to BH3 domain exposure [23,24] and/or to unleashing of a latent Bax-like pore-forming activity [25–27]. Yet a rigorous structural understanding of the conformational transitions in either Bax-type- or Bcl-2-type proteins responsible for exposure of the normally buried BH3 domain or for membrane permeabilization is currently lacking. Clearly, these observations beg further exploration of the structural properties of Bcl-2 family proteins in membrane environments.

Another exciting avenue for future research relates to the possible implication of OMM lipids in the function of Bcl-2 family proteins. Indeed, results obtained in model systems examining the lipid-dependence of Bax permeabilizing function lend support to a model whereby Bax permeabilizes the OMM by forming proteolipidic pores [9,28,29], akin to the mechanism of action proposed for colicins and other pore-forming toxins/peptides [30]. More recently, the levels of cardiolipin (and its degradation/oxidation derivatives) [31,32] and cholesterol [33,34] at the OMM level were implicated in regulating different stages of the Bax-driven membrane permeabilization pathway. Furthermore, functional coupling of tBid (but not Bim) with Bax in reconstituted systems involves specific tBid–cardiolipin interactions [35]. Based on these observations, it is reasonable to speculate that OMM lipids also play a role in the biological function of Bcl-2-type proteins. For example, Bcl-2-type proteins may interact with cardiolipin, cholesterol, or other OMM lipids that accumulate at the OMM during apoptosis [36] to control the Bax-driven membrane permeabilization pathway. Alternatively or in addition, the conformation and biological function of Bcl-2-type proteins themselves may be modulated via specific interactions with particular OMM lipids and/or through composition-dependent physical properties of the OMM bilayer. Simple model systems will provide a powerful tool with which to test the validity of these and other hypotheses on the mechanism of Bcl-2 protein action.

## Abbreviations

BH: Bcl-2 homology
OMM: outer mitochondrial membrane
tBid: cleaved/truncated Bid
